# Integrated bioinformatics analysis and experimental validation reveal ISG20 as a novel prognostic indicator expressed on M2 macrophage in glioma

**DOI:** 10.1186/s12885-023-11057-0

**Published:** 2023-06-28

**Authors:** Yaojun Peng, Hongyu Liu, Qiyan Wu, Lingxiong Wang, Yanju Yu, Fan Yin, Cong Feng, Xuewen Ren, Tianyi Liu, Ling Chen, Haiyan Zhu

**Affiliations:** 1 Department of Graduate Administration, Medical School of Chinese, PLA General Hospital, Beijing, China; 2 Department of Emergency, The First Medical Center, Chinese PLA General Hospital, 28Th Fuxing Road, Beijing, China; 3 Department of Neurosurgery, The First Medical Center, Chinese PLA General Hospital, 28Th Fuxing Road, Beijing, China; 4 Department of Neurosurgery, Hainan Hospital of Chinese PLA General Hospital, Sanya, Hainan China; 5 Institute of Oncology, The Fifth Medical Centre, Chinese PLA General Hospital, 8Th East Road of Fengtai, Beijing, China; 6 Department of Oncology, The Second Medical Center & National Clinical Research Center of Geriatric Disease, Chinese PLA General Hospital, Beijing, China

**Keywords:** ISG20, Glioma, Macrophage, Immune infiltration, Prognosis, Immune checkpoint

## Abstract

**Background:**

Glioma is the most common malignant primary brain tumor and is characterized by a poor prognosis and limited therapeutic options. ISG20 expression is induced by interferons or double-stranded RNA and is associated with poor prognosis in several malignant tumors. Nevertheless, the expression of ISG20 in gliomas, its impact on patient prognosis, and its role in the tumor immune microenvironment have not been fully elucidated.

**Methods:**

Using bioinformatics, we comprehensively illustrated the potential function of ISG20, its predictive value in stratifying clinical prognosis, and its association with immunological characteristics in gliomas. We also confirmed the expression pattern of ISG20 in glioma patient samples by immunohistochemistry and immunofluorescence staining.

**Results:**

*ISG20* mRNA expression was higher in glioma tissues than in normal tissues. Data-driven results showed that a high level of *ISG20* expression predicted an unfavorable clinical outcome in glioma patients, and revealed that ISG20 was possibly expressed on tumor-associated macrophages and was significantly associated with immune regulatory processes, as evidenced by its positive correlation with the infiltration of regulatory immune cells (e.g., M2 macrophages and regulatory T cells), expression of immune checkpoint molecules, and effectiveness of immune checkpoint blockade therapy. Furthermore, immunohistochemistry staining confirmed the enhanced expression of ISG20 in glioma tissues with a higher WHO grade, and immunofluorescence assay verified its cellular localization on M2 macrophages.

**Conclusions:**

ISG20 is expressed on M2 macrophages, and can serve as a novel indicator for predicting the malignant phenotype and clinical prognosis in glioma patients.

**Supplementary Information:**

The online version contains supplementary material available at 10.1186/s12885-023-11057-0.

## Introduction

Primary brain tumors are a heterogeneous group of tumors that arise from cells within the central nervous system (CNS) [1]. Gliomas represent 75% of the malignant primary brain tumors in adults [2]. The clinical management of glioma remains a significant challenge, as surgery and standard of care cytotoxic therapies (including radiation and chemotherapy) often offer minimal survival benefit [3]. Tumor heterogeneity, a hallmark of glioma, affects the genetic and epigenetic expression of specific cancer-related genes, modulation of metabolic pathways, and immune system evasion [4]. Notably, cell-to-cell crosstalk within the tumor microenvironment (TME) is recognized as a key player contributing to tumor heterogeneity; thus, facilitating malignant growth and immune evasion of glioma [5]. The glioma TME hosts a unique collection of cells, soluble factors, and extracellular matrix (ECM) components that regulate the evolution of glioma [6]. Macrophages and other myeloid cells are abundant in the brain TME and strongly correlate with aggressive phenotypes, distinct genetic signatures, cancer-induced immunosuppression, and responses to immunotherapies [6]. Therefore, the identification of immune-relevant biomarkers that reflect the functional status of macrophages in glioma is of great significance.

ISG20 was first discovered as a novel interferon (IFN)-regulated protein in Daudi cells in the year of 1997 [7]. It was revealed in the later studies that IFN regulatory factor 1 (IRF1) could govern the transcription of ISG20 in a type I (α/β) or a type II (γ) IFN dependent manner wherein a unique interferon-stimulated response element (ISRE) situated in the promoter region of ISG20 was stimulated [8–10]. The basal expression of ISG20 in various type of cells could also be regulated by different transcription factors, such as specificity protein 1 (SP-1) or upstream stimulatory factor 1 (USF-1); therefore, ISG20 could participate in the regulation of cellular functions in a IFN independent manner [8]. ISG20 was identified by Gongora et al. in breast cancer cell lines as a human estrogen-regulated transcript (HEM45); hence, it was also named ISG20/HEM45 [11]. ISG20 can cleave single-stranded RNA or DNA and is significantly associated with host antiviral innate immune defense [12, 13]. Several reports also suggest a link between ISG20 and the tumorigenic process of multiple neoplasms, including glioma [14], oral tumor [15], clear cell renal cell carcinoma [16], hepatocellular carcinoma [17], breast cancer [18], and acute myeloid leukemia [19], although the exact ISG20 pathomechanism remains unclear.

In the current study, we comprehensively illustrated the potential function of ISG20, its predictive value in stratifying clinical prognosis, and its association with immunological characteristics in glioma by adopting a bioinformatics methodology. We also confirmed the expression pattern of ISG20 in glioma patient samples by immunohistochemistry and immunofluorescence staining. Our study revealed that upregulation of ISG20 is positively correlated with unfavorable overall survival (OS) among patients with glioma. Enrichment analysis indicated that neuroactivity, ECM remodeling, immune response, and tumor immunity are associated with upregulated ISG20. Additionally, data-driven results suggested that ISG20 was possibly expressed on tumor-associated macrophages and was significantly associated with immune regulatory processes, as evidenced by its positive correlation with the infiltration of regulatory immune cells (e.g., M2 macrophages and regulatory T cells [Tregs]), expression of immune checkpoint molecules, and effectiveness of immune checkpoint blockade therapy. Finally, immunohistochemical staining showed upregulation of ISG20 in glioma tissues with a higher WHO grade, and the immunofluorescence assay verified that ISG20 was expressed in M2 macrophages. These data shed light on the cellular and molecular basis of the glioma immune microenvironment, thereby guiding the development of immunomodulatory strategies in gliomas.

## Methods

### TCGA glioma data acquisition

Normalized level 3 gene expression data and corresponding clinical information of TCGA glioma samples were downloaded from the UCSC Xena database (http://xena.ucsc.edu/). A total of 702 samples were acquired, including 5 normal brain tissues and 697 glioma tissues (530 cases of LGG and 167 cases of GBM). The clinical information of the glioma samples is summarized in Table S1. The expression levels of *ISG20* in normal human tissues from GTEx and pan-cancer expression of *ISG20* across TCGA tumors were extracted from the UCSC Xena database. The abbreviations for TCGA tumors are listed in Table S2.

### ISG20 gene expression analysis

The Gene Expression database of Normal and Tumor tissues 2 (GENT2) database (http://gent2.appex.kr/gent2/) is a user-friendly search platform for gene expression patterns across different normal and tumor tissues compiled from public gene expression datasets deposited in the Gene Expression Omnibus database [20]. The expression of *ISG20* in human tumors and normal tissues across different cancers was assessed using GENT2. For glioma, the expression of *ISG20* was extracted from the TCGA glioma dataset and analyzed in diverse clinical statuses, including age, sex, IDH mutation, 1p19q codeletion, MGMT methylation, grade, histology, and primary therapy outcome. The single-cell expression profile of ISG20 in human brain tissue was retrieved from the Human Protein Atlas (HPA) online database (http://www.proteinatlas.org).

### Survival analysis

The GENT2 database also provides reliable prognostic power estimated by meta-survival analysis across many independent reports, allowing integrated statistical analysis from different studies, increasing the number of samples, and improving statistical power [20]. The association between *ISG20* expression and the OS of patients with brain tumors was analyzed by meta-survival analysis using the GENT2 database. The prognostic value of ISG20 in glioma was further explored in TCGA glioma patients using the Kaplan–Meier method and log-rank test. Time-dependent ROC analysis was also used to estimate the prognostic value of *ISG20* for survival prediction in patients with TCGA glioma.

### Association between *ISG20* expression and immunological characteristics

The ESTIMATE score provides researchers with scores for tumor purity, the level of stromal cells that are present, and the infiltration level of immune cells in tumor tissues based on expression data. The stromal, immune, and ESTIMATE scores for TCGA glioma samples were retrieved from the ESTIMATE website maintained by the MD Anderson Cancer Center (https://bioinformatics.mdanderson.org/estimate/) and compared between the glioma patient subgroups classified by the median expression level of *ISG20*.

The putative immune cell infiltration of TCGA glioma patients was retrieved from the TIMER2.0 website (http://timer.comp-genomics.org/), a comprehensive resource that contains 10,897 samples across 32 cancer types from TCGA and is a powerful tool for systematic analysis of immune infiltrates across diverse cancer types [21]. The abundance of immune cells was compared between the glioma patient subgroups classified according to the median expression level of *ISG20*. Moreover, the correlation between *ISG20* expression levels and the abundance of immune cells was calculated using Spearman’s correlation analysis.

### Association between *ISG20* expression and response of immunotherapy

The immunophenoscore (IPS) of TCGA GBM patients was downloaded from the Cancer Immunology Atlas (TCIA, https://tcia.at/patients) [22]. The patient’s IPS was obtained without prejudice by considering four types of immunogenic determinants: effector cells, immunosuppressive cells, MHC molecules, and immunomodulators. This step was performed by evaluating gene expression in the four cell types. The IPS is calculated based on the z-score representing gene expression in the cell type in the range of 0–10. A higher IPS score was positively correlated with increased immunogenicity. The Wilcoxon rank-sum test was used to compare the differences in the IPS scores between the high and low *ISG20* expression subgroups.

### Glioma sample collection, immunohistochemistry and immunofluorescence

This study was conducted in accordance with the Declaration of Helsinki and was approved by the Institutional Research Ethics Committee of the PLA General Hospital. Signed informed consent was obtained from all the participants. A total of 28 paraffin-embedded glioma samples were used for immunohistochemistry and immunofluorescence staining. The clinical information of the glioma samples is shown in Table S3.

Immunohistochemistry was performed to examine ISG20 and CD163 expression in serial sections from glioma patients. Formalin-fixed and paraffin-embedded tissue specimens were deparaffinized and subjected to heat-induced epitope retrieval in citrate buffer solution. The slices were then blocked with 5% bovine serum albumin for 30 min and incubated with rabbit anti-ISG20 antibody (1:1000, Proteintech, Wuhan, China) or mouse anti-CD163 monoclonal antibodies (1:500, Gene Tech, Shanghai, China) at 4 °C overnight, followed by incubation with a secondary antibody for 90 min at 37 °C. Detection was achieved with 3,3′-diaminobenzidine (ZSGB-BIO, Beijing, China), counterstained with hematoxylin, dehydrated, cleared, and mounted as in routine processing. Protein expression level was quantified by the immunoreactivity score (IRS) calculated as IRS (0–12) = RP (0–4) × SI (0–3), where RP represents the percentage of staining-positive cells and SI is the staining intensity.

To estimate the density of ISG20 expression in M2-type tumor-associated macrophages, an immunofluorescence assay was performed. Formalin-fixed and paraffin-embedded tissue specimens were deparaffinized and subjected to heat-induced epitope retrieval in citrate buffer solution. Subsequently, the sections were blocked with goat serum containing 0.3% Triton at room temperature for 30 min. Rabbit anti-ISG20 polyclonal antibody (1:1000, Proteintech, Wuhan, China) and mouse anti-CD163 monoclonal antibodies (1:500, Gene Tech, Shanghai, China) were used, followed by Alexa Fluor 488-conjugated (1:400, Abcam, Boston, MA, USA) anti-rabbit antibody and Alexa Fluor 568-conjugated anti-mouse antibody (1:400, Abcam, Boston, MA, USA). Images were captured using a confocal laser-scanning microscope (Olympus FV1000). The acquired images were further processed and analyzed using ImageJ software (version 1.8.0).

### Pathway enrichment analysis

Differentially expressed genes (DEGs) between the *ISG20* low and high subgroups (classified by the median expression of *ISG20*) were identified using R software (version 4.1.2) with limma package, and the screening criteria were set as log_2_ |fold change|≥ 1 and adjusted *P*-value < 0.05. These DEGs were subjected to Gene Ontology (GO) and Kyoto Encyclopedia of Genes and Genomes (KEGG) [23] analyses using Metascape [24], a free online tool for gene annotation (http://metascape.org). Functional annotation of GO was categorized into three major categories: Biological Process (BP), Cellular Component (CC), and Molecular Function (MF). GO or KEGG terms with *P*-value < 0.01 were considered significantly enriched. Gene set enrichment analysis (GSEA) [25] was used to determine whether members of a given gene set were generally associated with ISG20. The expression level of ISG20 (high or low) was designated as the phenotype, and analysis was conducted using the matched gene expression profile. Random sample permutations and the significance threshold were set at 1000 times and false discovery rate < 0.05. GSEA was performed using the JAVA program (http://software.broadinstitute.org/gsea/index.jsp) using h.all.v7.4. symbols.gmt gene set collection downloaded from the Molecular Signatures Database (http://www.gsea-msigdb.org/gsea/downloads.jsp) was used as an annotation reference. The enriched pathways were ranked by enrichment score. If a gene set had a positive enrichment score, the high expression level of the majority of its members was positively related to the *ISG20* high phenotype.

### Statistical analysis

For bioinformatics analysis, the Wilcoxon rank-sum test was used to compare the differences between two groups, and the comparison of multiple groups was performed using the Kruskal–Wallis test and Dunn’s t-test. The correlation between ISG20 expression and other relevant genes or the abundance of putative infiltrating immune cells was evaluated using Spearman’s correlation analysis. When analyzing the results of immunohistochemistry, Student’s *t*-test was used to compare the differences in IRSs. Statistical analyses were performed using R software (version 3.6.3) or GraphPad Prism (version 9.0.0), and *P* values < 0.05 were considered statistically significant. All statistical tests were two sided.

## Results

### Elevated *ISG20* expression in glioma

**Fig. 1:**
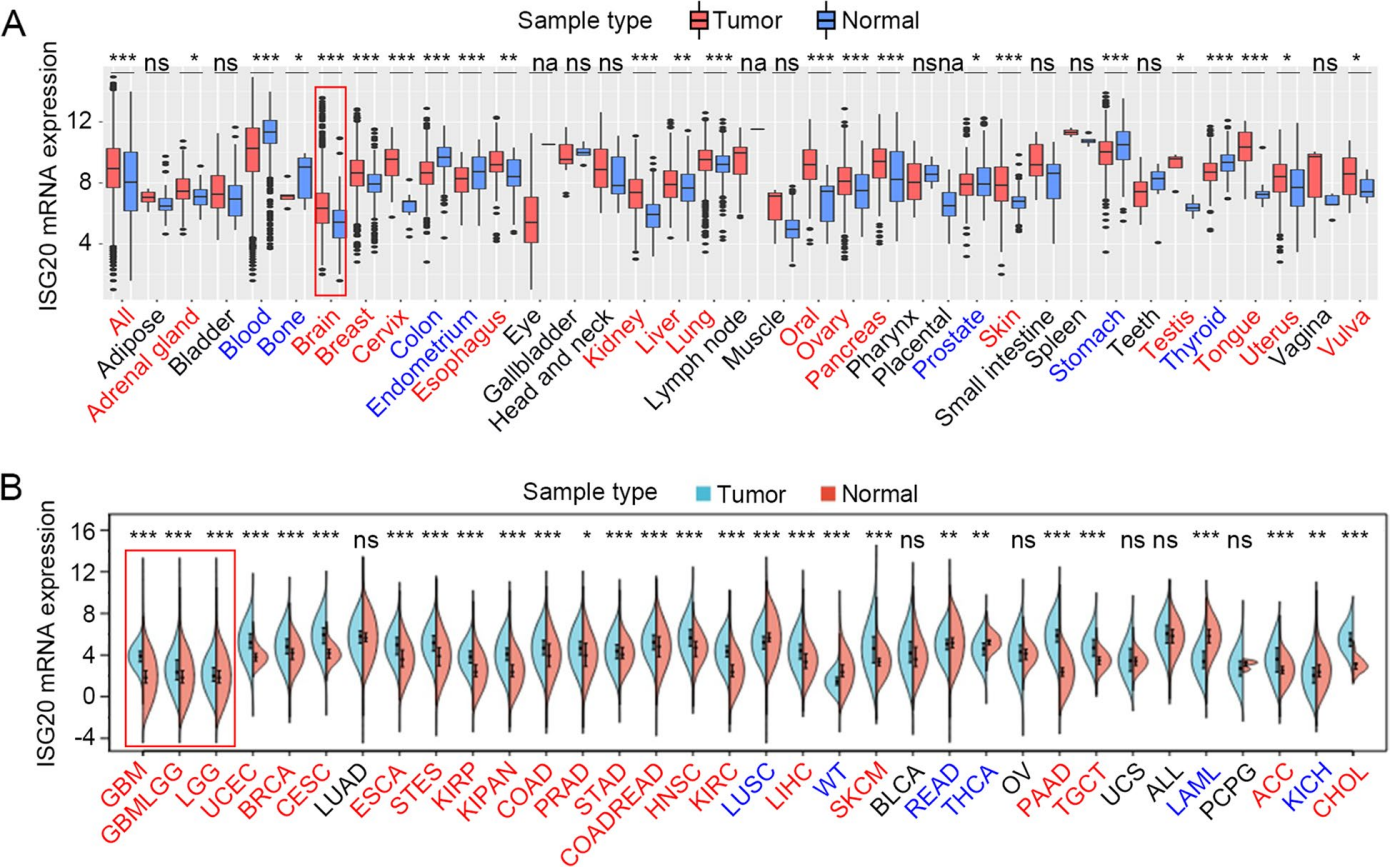
*ISG20* mRNA expression level in tumors and normal tissues. **A** *ISG20* mRNA expression in tumors and normal tissues in microarray datasets collected in GENT2 database. **B** *ISG20* mRNA expression in TCGA tumors and normal tissues with the GTEx database as controls. na: not available. ns: no significance, *P < 0.05, **P < 0.01, ***P < 0.001

We explored the transcriptional expression of *ISG20* in human cancer and normal samples using microarray data from the GENT2 database. In general, the expression of *ISG20* was higher in cancer samples than in normal samples (*P* < 0.001; Fig. 1A). *ISG20* expression was lower in normal tissues than in neoplastic sites across the majority of cancer types, including adrenal gland, brain, breast, cervix, esophagus, kidney, liver, lung, oral, ovary, pancreas, skin, testis, tongue, uterus, and vulvar tumors. In contrast, decreased expression of *ISG20* was observed in the blood, bone, colon, endometrium, prostate, stomach, and thyroid tumors (Fig. 1A). We further validated the expression levels of *ISG20* in human cancer and normal samples using RNA-seq data derived from the TCGA and GTEx databases. The results showed that ISG20 was uniformly overexpressed in brain, uterus, breast, cervix, esophagus, kidney, liver, pancreas, skin, and testis tumors across the GENT2 and TCGA databases (Fig. 1B). In particular, glioma tissues (LGG and GBM) uniformly expressed higher levels of *ISG20* than normal brain tissues did, and the difference was most significant in GBM, the most aggressive subtype of glioma (Fig. 1B).

### Increased *ISG20* expression is correlated to malignant phenotypes of glioma

**Fig. 2:**
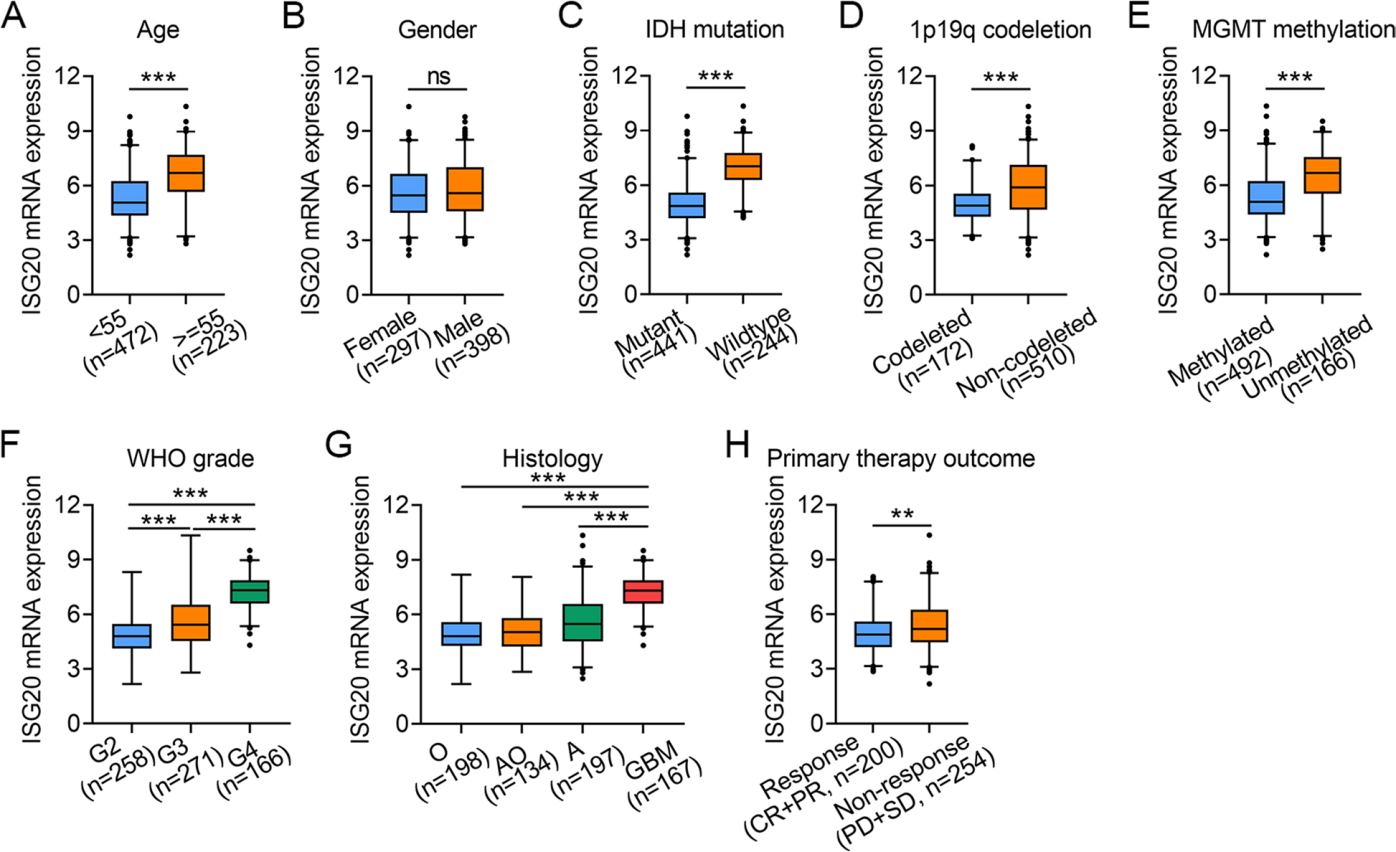
Associations between *ISG20* mRNA expression and different clinical characteristics of glioma patients. **A** Age. **B** Gender. **C** IDH mutation status. **D** 1p19q codeletion. **E** MGMT methylation. **F** WHO grade. **G** Histology. **H** Primary therapy outcome. *O* Oligodendroglioma, *AO* Anaplastic oligodendrocytoma, *A* Astrocytoma, *GBM* Glioblastoma, *CR* Complete response, *PR* Partial response, *PD* Progressive disease, *SD* Stable disease. *ns* no significance, **P < 0.01, ***P < 0.001

To further explore the expression pattern of *ISG20* in glioma, we analyzed the expression of *ISG20* in patient subgroups with disparate clinical characteristics, including age, gender, IDH mutation, 1p19q co-deletion, MGMT methylation, WHO grade, histology, and primary therapy outcome. Our data revealed an increase in *ISG20* expression in patients older than 55 years (*P* < 0.001; Fig. 2A), whereas *ISG20* was not differentially expressed between males and females (*P* > 0.05; Fig. 2B). Regarding IDH mutation status, *ISG20* expression was markedly enhanced in glioma tissues with wild-type IDH (*P* < 0.001; Fig. 2C**)**. In terms of 1p19q codeletion status, upregulation of *ISG20* was noted in glioma tissues with 1p19q non-codeletion (*P* < 0.001; Fig. 2D). Regarding MGMT methylation status, *ISG20* was overexpressed in glioma tissues with unmethylated MGMT (*P* < 0.001; Fig. 2E). Stratifying by WHO grade, ISG20 expression was highest in G4 gliomas, followed by G3 and G2 gliomas (*P* < 0.001; Fig. 2F). Additionally, *ISG20* expression was significantly higher in glioblastoma, followed by astrocytoma, anaplastic oligodendrocytoma, and oligodendroglioma (*P* < 0.001; Fig. 2G). We also observed that the expression of *ISG20* was enhanced in patients who were unresponsive to routine therapy (*P* < 0.01; Fig. 2H). These results suggest that high *ISG20* expression is positively correlated with the malignant phenotype of gliomas and marginal therapeutic efficacy.

### *ISG20* overexpression is associated with unfavorable prognosis of patients with glioma

**Fig. 3:**
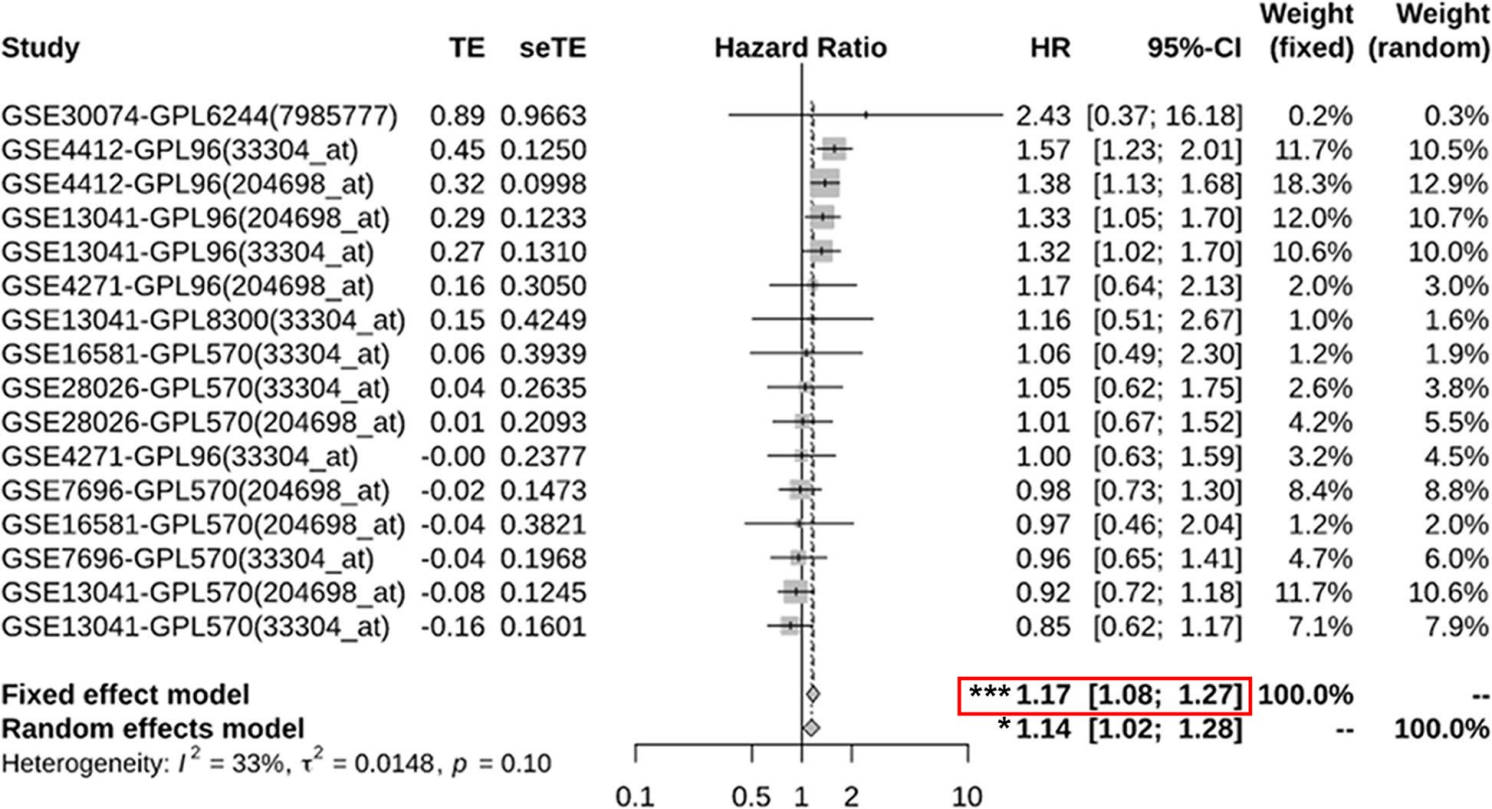
Meta-survival analysis of *ISG20* in brain tumor patients in GENT2 database. *TE* Estimate of treatment effect, *seTE* Standard error of treatment estimate, *HR* Hazard ratio, *CI* Confidence interval. *P < 0.05, ***P < 0.001

Using GENT2 which provides reliable prognostic power estimated by the synergetic effect across many independent reports, we first performed a meta-survival analysis of *ISG20* in brain tumors with differing histopathological types. A total of 7 individual brain tumor reports (GSE30074: medulloblastoma, GSE16581: meningioma, GSE28026: atypical teratoid/rhabdoid tumors, GSE4271: high-grade astrocytomas, GSE4412: gliomas, GSE13041 and GSE7696: GBM) were collected, and the forest plot of the hazard ratio of *ISG20* with OS in brain tumor patients is shown in Fig. 3. The fixed effect model of meta-survival analysis showed that *ISG20* overexpression was associated with unfavorable prognosis of patients with brain tumor (*P* < 0.001, HR = 1.17, 95% CI: 1.08–1.27; Fig. 3).

**Fig. 4:**
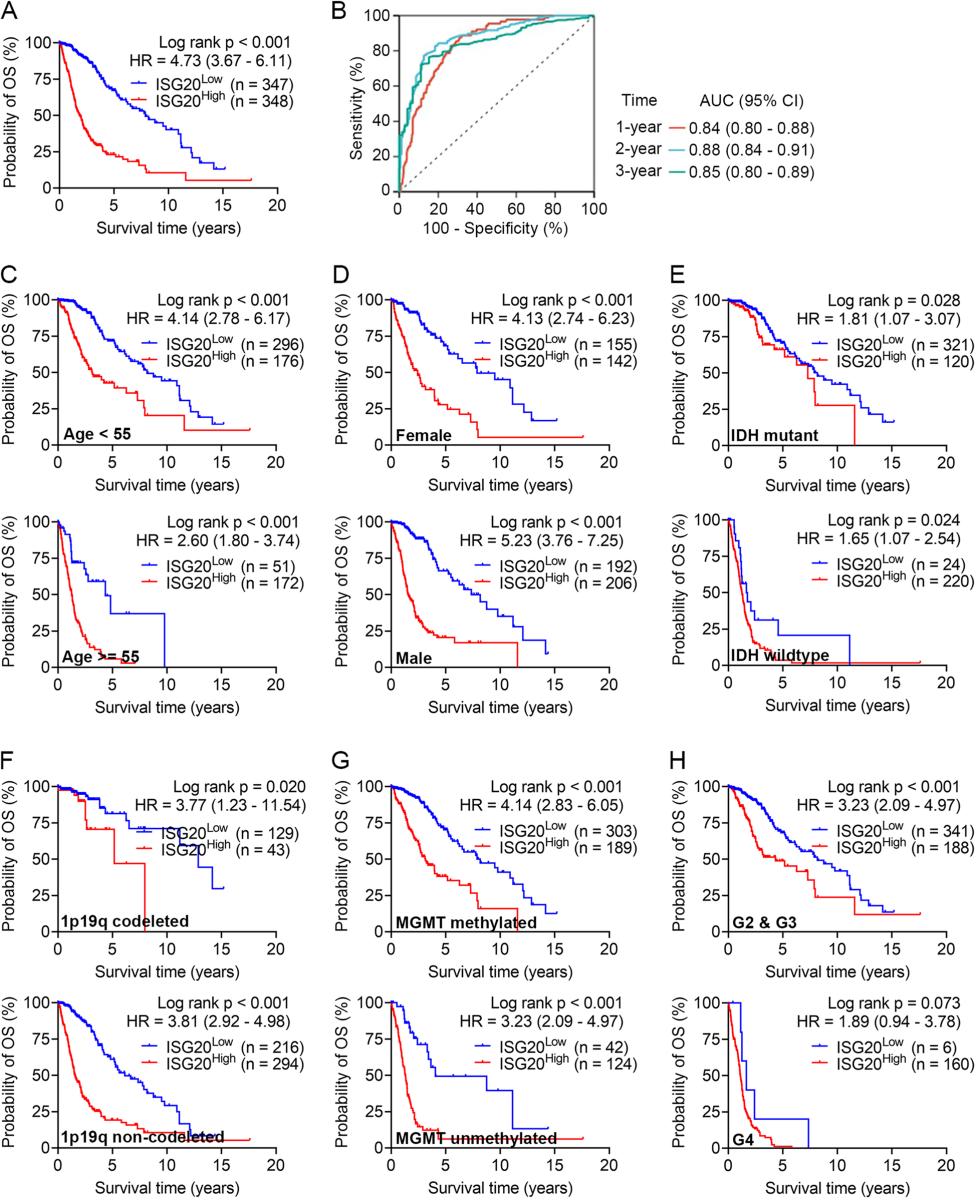
Prognostic value of the *ISG20* mRNA expression in glioma patients. **(A)** Survival curves of TCGA glioma patients stratified by *ISG20* mRNA expression. **(B)** Time-dependent ROC curves for *ISG20* expression in TCGA glioma patients. Stratification analysis of *ISG20* on patient survival in subgroups of glioma patients classified by **(C)** Age, **(D)** Gender, **(E)** IDH mutation, **(F)** 1p19q codeletion, **(G)** MGMT methylation, and **(H)** WHO grade. *HR* Hazard ratio, *CI* Confidence interval

To be more specific, we further explored the prognostic value of *ISG20* in patients with glioma by analyzing the TCGA glioma dataset. Kaplan–Meier survival analysis showed that glioma patients with elevated *ISG20* expression presented with unfavorable OS (*P* < 0.001, HR = 4.73, 95% CI: 3.67–6.11; Fig. 4A). Moreover, the time-dependent ROC analysis revealed that the *ISG20* expression had a relatively good performance in predicting 1-year (AUC = 0.84, 95% CI: 0.80–0.88), 2-year (AUC = 0.88, 95% CI: 0.84–0.91), and 3-year OS (AUC = 0.85, 95% CI: 0.80–0.89) in glioma patients (Fig. 4B). Furthermore, the stratification analysis showed that high *ISG20* expression may predict unfavorable OS in glioma patient subgroups with different age, sex, IDH mutation, 1p19q codeletion, and MGMT methylation (all *P* < 0.05; Fig. 4C-G). As for WHO grade, the *ISG20* expression could identify patients with different prognoses in G2 and G3 subgroup (*P* < 0.001; Fig. 4H, upper panel), while the P value was not significant in G4 subgroup (*P* = 0.07; Fig. 4H, lower panel). This might be attributed to the small sample size of *ISG20* low glioma patients (*n* = 6) in the G4 subgroup to draw any reliable conclusions. Taken together, the above analyses suggest that a higher *ISG20* expression level is correlated with a worse prognosis in glioma patients.

### Exploring the signaling pathways related to *ISG20* in glioma

**Fig. 5:**
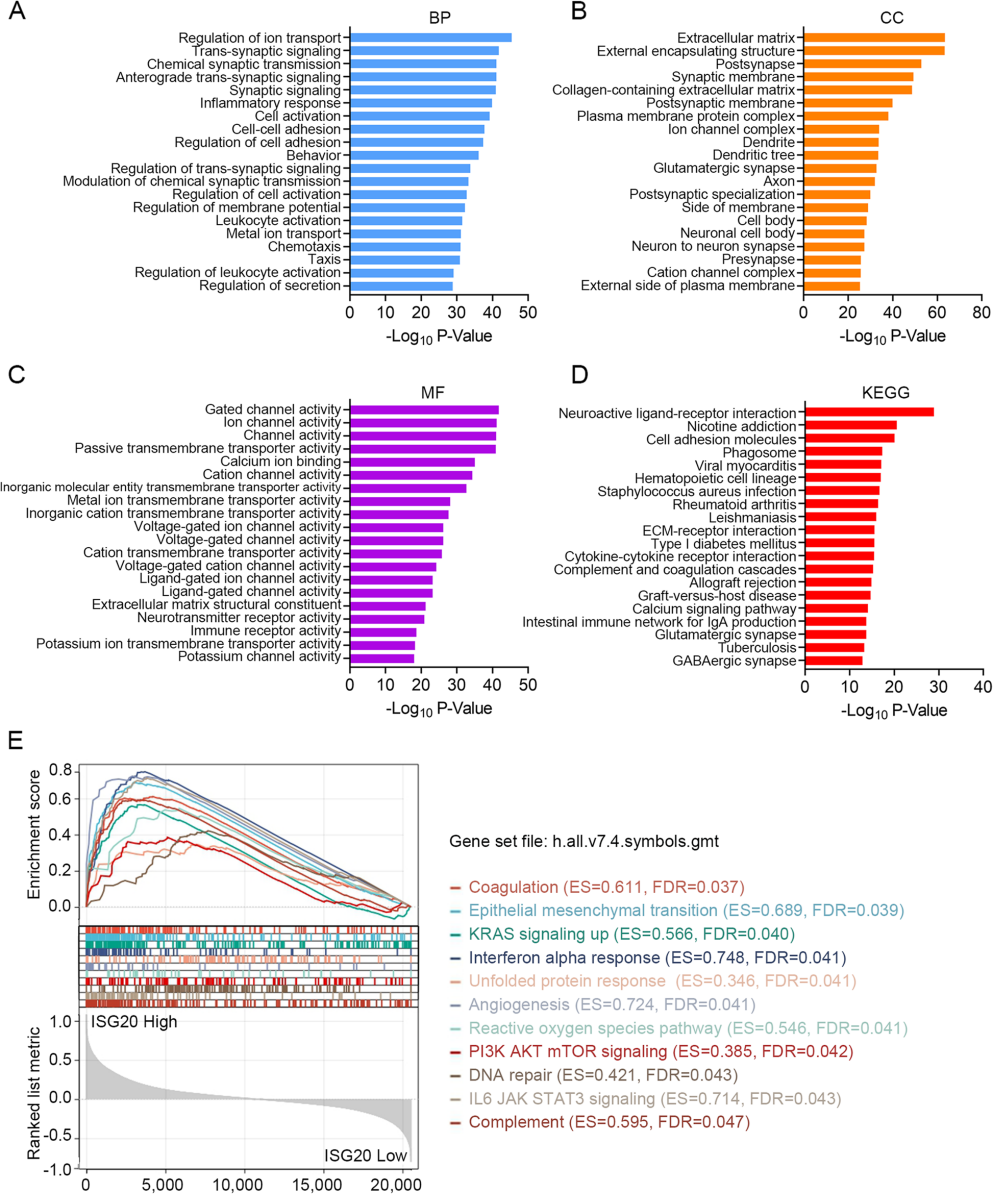
Exploring the signaling pathways related to ISG20 in glioma. Top 20 enriched GO terms (**(A)** Biological process, **(B)** Cellular component, and **(C)** Molecular function) by the differential expressed genes between *ISG20* low and high subgroups. **(D)** Top 20 enriched KEGG terms. **(E)** GSEA analysis showed significantly enriched pathways in glioma patients using *ISG20* expression as phenotype (*ISG20* low *vs. ISG20* high). *BP* Biological process, *CC* Cellular component, *MF* Molecular function, *ES* Enrichment score, *FDR* False discovery rate

To investigate the underlying mechanisms of *ISG20* in glioma, functional enrichment analyses were performed based on DEGs between patients with high or low expression levels of *ISG20*. There were 2,624 DEGs between the glioma patient subgroups classified according to the median expression level of *ISG20*, of which 1,392 were upregulated and 1,232 were downregulated in the subgroup expressing higher levels of *ISG20* (Figure S1 and Table S4). These DEGs were subjected to Metascape database to identify functional GO and KEGG terms. As for BP, the DEGs were mainly enriched in items such as regulation of ion transport, synaptic signaling, cell activation, cell adhesion, and inflammatory response (Top 20; Fig. 5A). In terms of CC, these genes were mainly involved in the ECM, synaptic/postsynaptic membrane, and neuronal cell body (Top 20; Fig. 5B). Regarding MF, these genes mainly participated in multiple channel activities, ECM structural activity, neurotransmitter receptor activity, and immune receptor activity (Top 20; Fig. 5C). In addition, KEGG analysis revealed that these DEGs were mainly associated with neuroactive ligand-receptor interactions, cell adhesion molecules, ECM-receptor interactions, cytokine-cytokine receptor interactions, and complement and coagulation cascades (Top 20; Fig. 5D). The complete lists of the enriched GO and KEGG terms are shown in Table S5, S6, S7, S8. Additionally, GSEA was performed to identify *ISG20* related biological functions in gliomas. Accordingly, *ISG20* upregulation was associated with coagulation, epithelial-mesenchymal transition, angiogenesis, complement, and cancer/immune-related signaling, such as KRAS, PI3K-AKT-mTOR, and IL6-JAK-STAT3 (Fig. 5E). Collectively, these results highlight the functions of *ISG20* in neuroactivity, ECM remodeling, immune response, and tumor immunity, allowing us to revisit its immunological role in subsequent analyses.

### Association between *ISG20* expression and immune cell infiltration of glioma

**Fig. 6:**
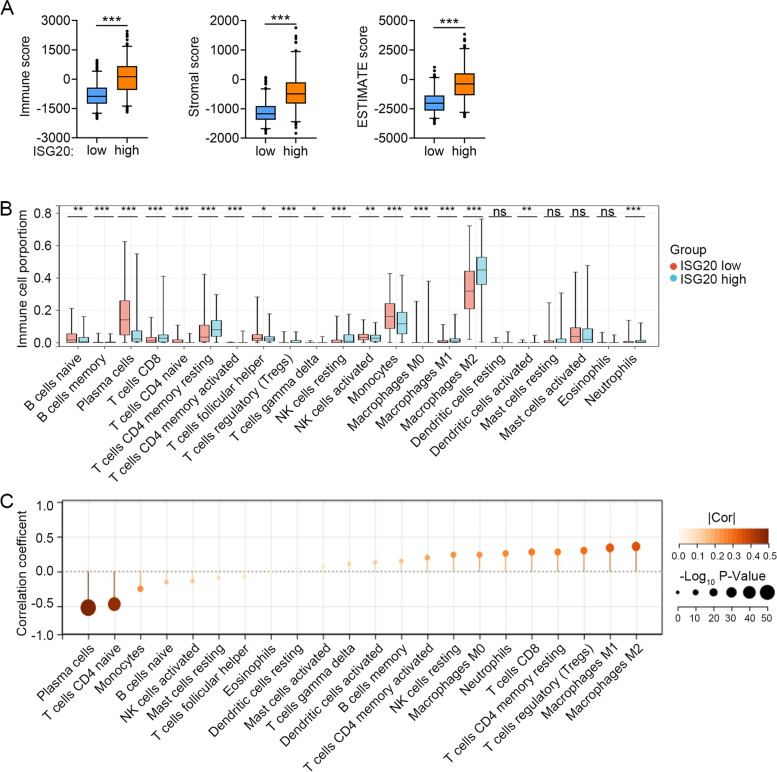
*ISG20* mRNA expression level was associated with unique immune microenvironment in glioma. **A** Comparison of Immune score, Stromal score, and ESTIMATE score between *ISG20* low and high groups. **B** Box plots depicting the abundance of 22 immune cells of the *ISG20* high group compared to ISG20 low group. **C** Correlation between *ISG20* expression and abundance of 22 immune cells. ns: no significance, *P < 0.05, **P < 0.01, ***P < 0.001

These data showed that *ISG20* was correlated with prognosis and tumor immunity in glioma; therefore, we explored the role of *ISG20* in TME remodeling and immune cell regulation. Analysis of tumor purity, the level of stromal cells that are present, and the infiltration level of immune cells resolved by ESTIMATE revealed that glioma samples with enhanced *ISG20* expression manifested increased immune, stromal, and ESTIMATE scores (all *P* < 0.001; Fig. 6A). Specifically, putative immune cell infiltration was estimated using the CIBERSORT algorithm and compared between the glioma patient subgroups classified by the median expression level of *ISG20*. The results indicated that M2 macrophages, M1 macrophages, Tregs, M0 macrophages, CD4^+^ memory resting T cells, CD8^+^ T cells, neutrophils, resting NK cells, memory B cells, activated CD4^+^ memory T cells, activated myeloid dendritic cells, and gamma delta T cells were enriched (all *P* < 0.05; Fig. 6B), whereas the abundance of plasma cells, CD4^+^ naïve T cells, monocytes, naïve B cells, activated NK cells, and follicular helper T cells was reduced in the *ISG20* high subgroups (all *P* < 0.05; Fig. 6B). We also performed correlation analysis to infer the relationship between *ISG20* expression and immune cell infiltration. As shown in Fig. 6C, these results further support the above findings, as shown by the strong correlations between *ISG20* expression and M2 macrophages (*r* = 0.36, *P* < 0.001), M1 macrophages (*r* = 0.34, *P* < 0.001), Tregs (*r* = 0.30, *P* < 0.001), plasma cells (*r* = -0.52, *P* < 0.001), and CD4^+^ naïve T cells (*r* = -0.47, *P* < 0.001). Taken together, these results suggest that *ISG20* expression might predict the immunosuppressive status of the TME and immune cell infiltration in gliomas, especially macrophage and T cell immune responses.

**Fig. 7:**
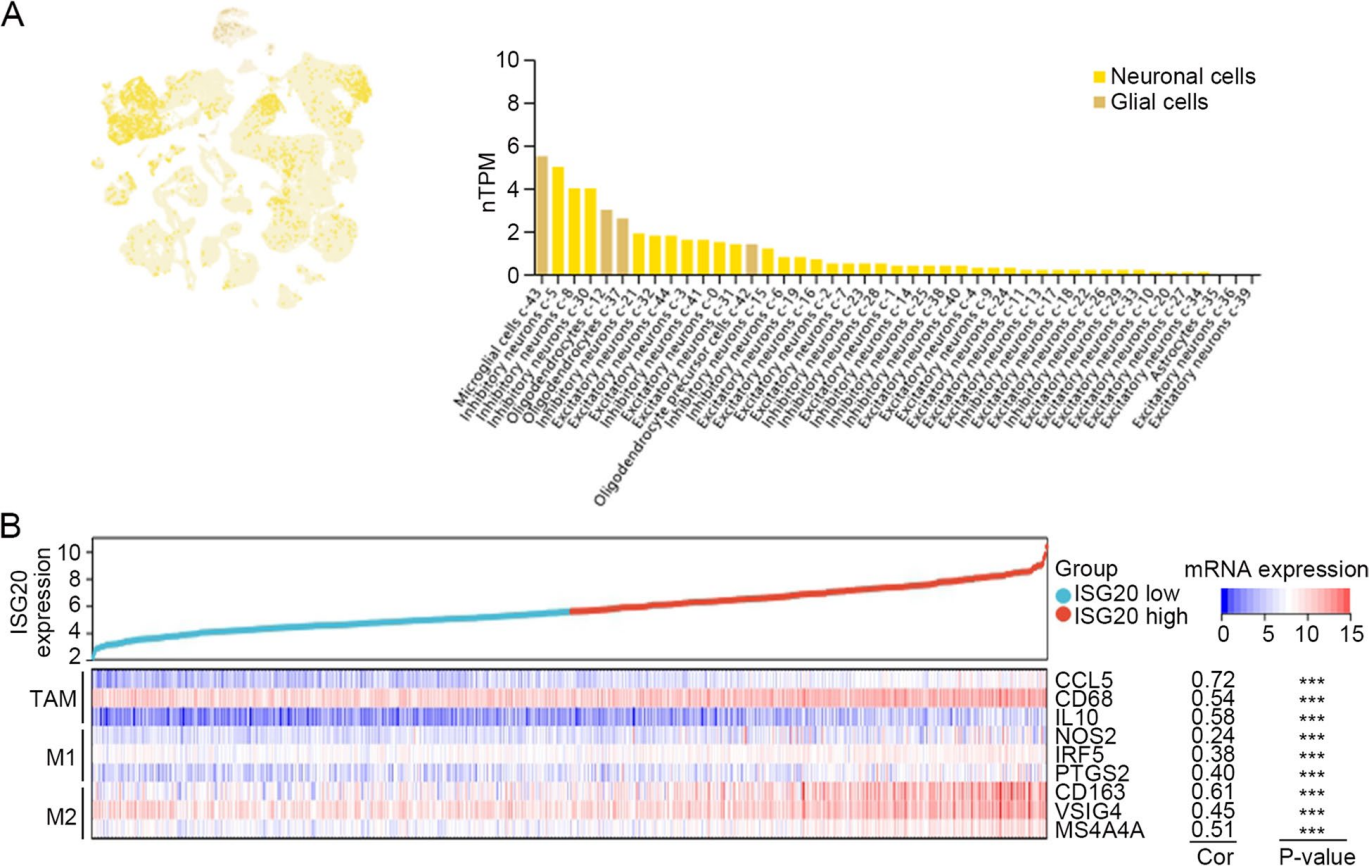
*ISG20* was expressed in tumor-associated macrophages in glioma. **A** Expression of *ISG20* in different types of cells in normal brain tissues by single-cell transcriptional analysis in the HPA database. **B** Correlations between *ISG20* and marker genes of tumor-associated macrophages, and M1 and M2 macrophages

In correlation analysis, we observed the most positive correlation between M2 macrophage infiltration and *ISG20* expression, prompting us to gain insight into the cellular distribution of *ISG20* on macrophages and its role in macrophage polarization in gliomas. Single-cell transcriptome-based analysis using the HPA database revealed that microglial cells had the highest expression level of *ISG20* in human brain tissues (Fig. 7A). Microglia are highly versatile resident macrophages in the CNS that can be polarized into M1 and M2 phenotypes in response to diverse environmental stimuli [26]. We further explored the relationship between *ISG20* and the expression of marker genes of tumor-associated macrophages (TAMs) and M1 and M2 macrophages in TCGA glioma. The results showed that *ISG20* strongly correlated with the marker genes of TAMs and M2 macrophages (all *r* > 0.40, all *P* < 0.001; Fig. 7B). These findings suggest a possible participation of *ISG20* in the regulation of macrophage polarization in gliomas.

### Correlation between *ISG20* and immune checkpoint genes in glioma

**Fig. 8:**
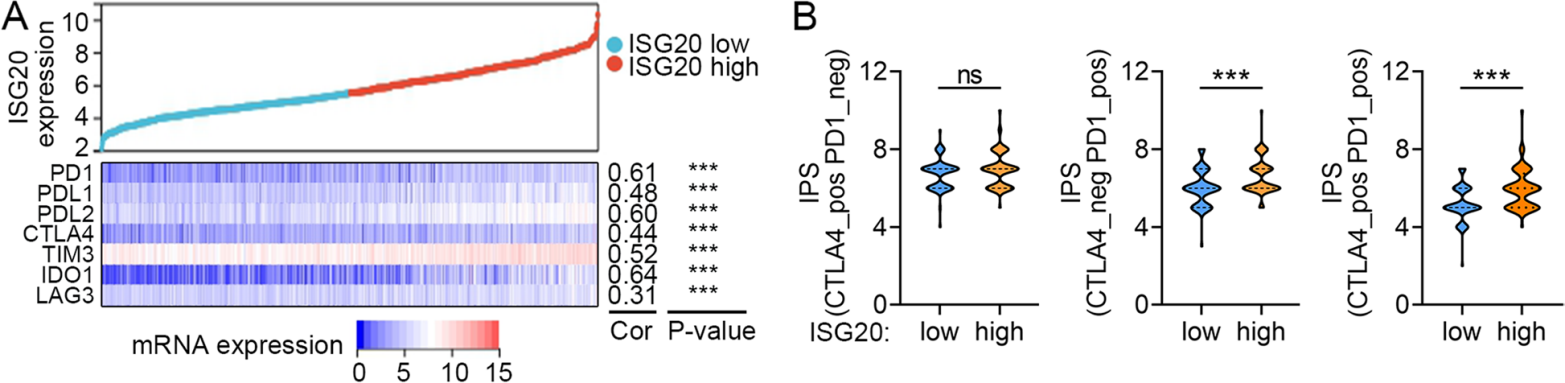
Estimation of association between *ISG20* mRNA expression with immunotherapy response. **A** Correlation of *ISG20* expression with immune checkpoints in TCGA glioma. **B** Effectiveness of CTLA4, PD1 and CTLA4 plus PD1 immunotherapy in *ISG20* low and high TCGA GBM patients

To better understand the immune-modulating functions of *ISG20* in glioma, we estimated the correlation between *ISG20* and a panel of immune checkpoint molecules, including *PD1*, *PDL1*, *PDL2*, *CTLA4*, *TIM3*, *IDO1*, and *LAG3*. *ISG20* was significantly associated with these immune checkpoint genes (all *r* > 0.30, *P* < 0.001; Fig. 8A), suggesting an immunoregulatory role of *ISG20* in the glioma immune microenvironment. We further investigated the variation between ISG20 expression and immunotherapy in TCGA GBM patients using the TICA database. The results showed that PD1 blockage and *PD1* plus *CTLA4* dual blockage had better treatment effectiveness in the *ISG20* high subgroup (both *P* < 0.001; Fig. 8B), whereas no significant difference in treatment effectiveness was observed regarding *CTLA4* blockage (*P* = 0.19; Fig. 8B). Taken together, these data showed that *ISG20* expression may be an indicator of *PD1* blockade treatment in GBM.

### Confirming the expression pattern of ISG20 in glioma tissues

**Fig. 9:**
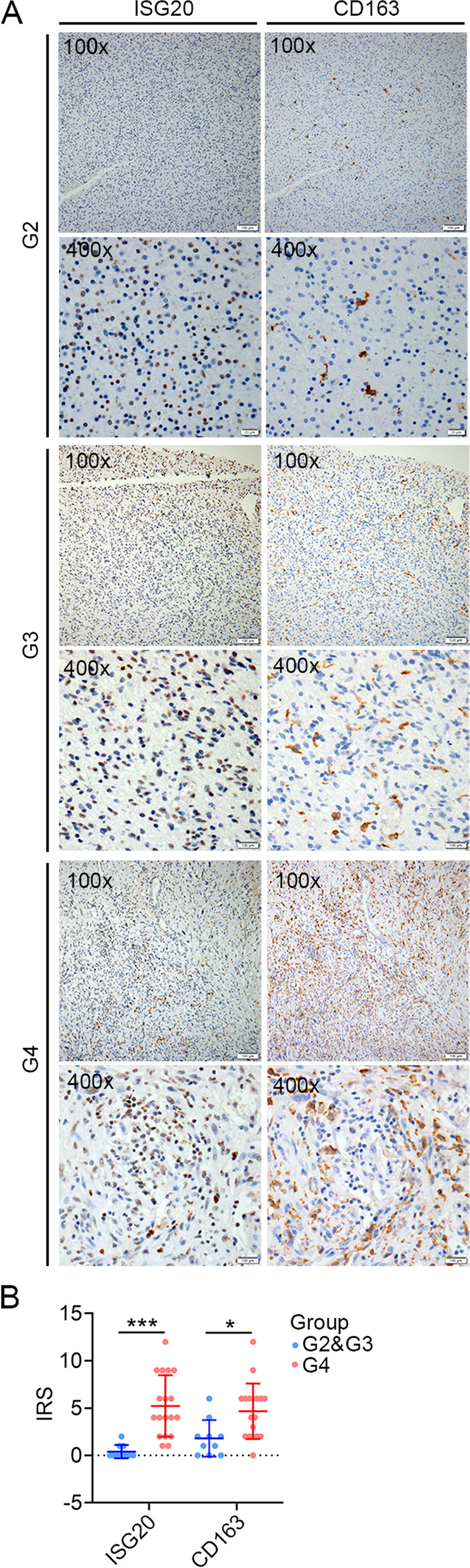
Immunohistochemistry staining of ISG20 and CD163 protein expression in serial sections from glioma specimens with different grades. **A** Representative immunofluorescence image of ISG20 and CD163 expression in WHO G2, G3, and G4 glioma. **B** Quantitative analysis of ISG20 and CD163 expression in low-grade (G2 and G3) and high-grade (G4) glioma specimens. IRS: immunoreactivity score. *P < 0.05, ***P < 0.001

Finally, we verified the protein expression pattern of ISG20 in clinical glioma specimens. ISG20 protein in situ expression was analyzed using immunohistochemical staining. ISG20 protein expression was higher in high-grade glioma (G4) than in low-grade glioma (G2 and G3), consistent with the results of the transcriptional analyses (*P* < 0.05; Fig. 9A and B). CD163 has been recognized as a well-known marker of M2 macrophages. We performed immunohistochemical staining of CD163 in the sister slices of those stained with ISG20. The results revealed that compared with low-grade gliomas, the intensity of CD163 protein in high-grade gliomas was also enhanced (*P* < 0.05), and there was a good match between ISG20 and CD163 expression in the serial sections (Fig. 9A and B).

**Fig. 10:**
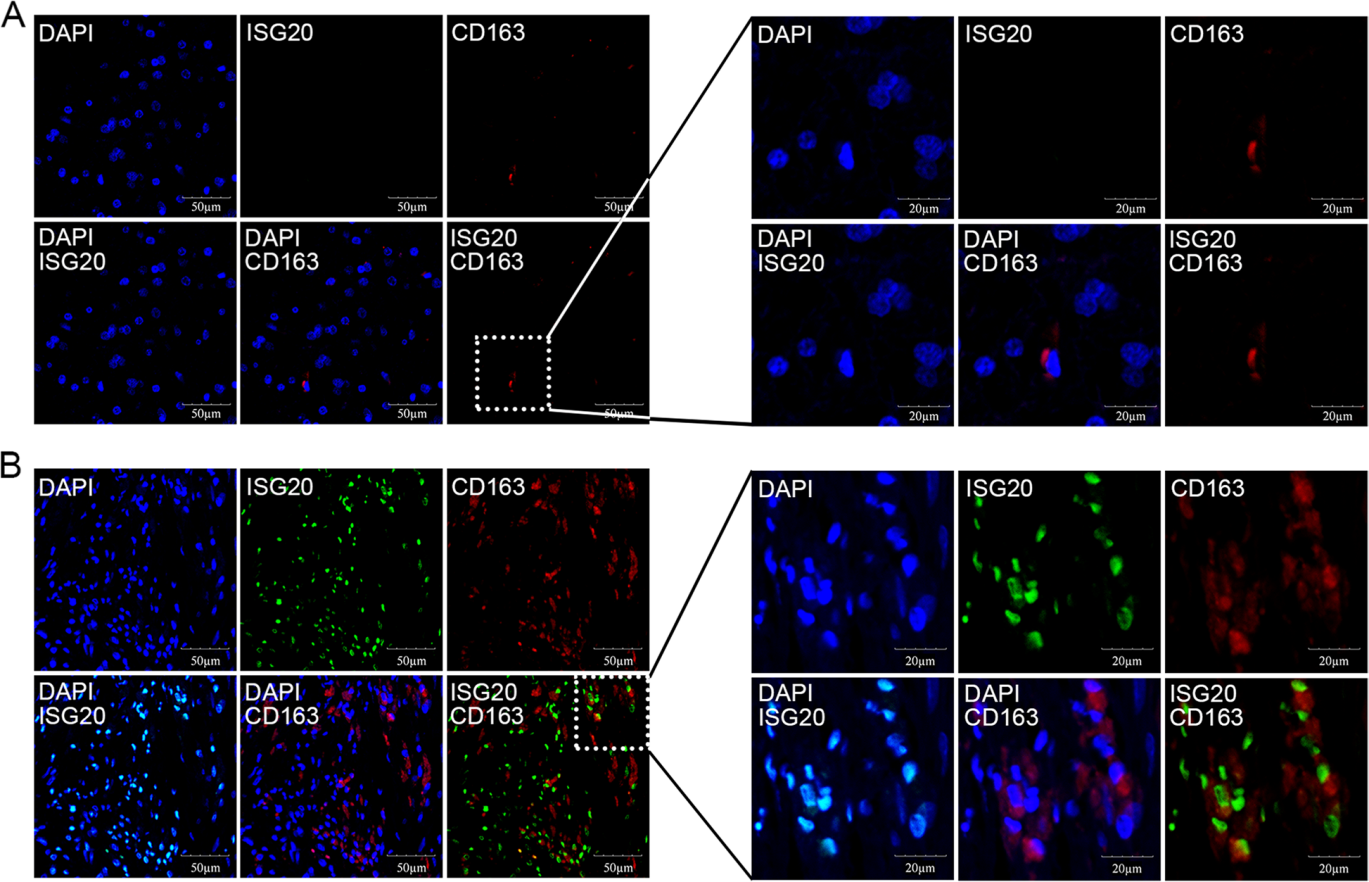
Immunofluorescence staining showing colocalization of ISG20 and CD163. Expression of ISG20 (green) and CD163 (red) in glioma specimens with low **(A)** or high **(B)** ISG20 expression. DAPI (blue) was used for nuclear staining

Immunofluorescence assay was performed to further explore the cellular localization of ISG20 and CD163 in glioma samples. The results revealed that ISG20 was substantially colocalized with CD163 (Fig. 10A and B).

## Discussion

Glioma is the most common aggressive and lethal tumor in the CNS, and is the predominant brain primary malignancy [27]. Despite tremendous progress in the diagnosis and management of glioma, the clinical prognosis of patients with glioma is dismal, with a 5-year OS of no greater than 35% [28]. Therefore, it is crucial to identify feasible cell type-specific biomarkers and to uncover the underlying mechanisms that contribute to the malignant phenotype of glioma. In the current study, we found that *ISG20* mRNA expression was significantly higher in gliomas than in normal tissues. Elevated ISG20 expression is associated with the malignant phenotype of glioma and marginal therapeutic efficacy. We also showed that a high level of *ISG20* expression was significantly associated with poor OS in glioma patients, as strengthened by stratification analyses in patient subgroups with differing age, sex, IDH mutation status, 1p19q codeletion status, MGMT methylation, and WHO grade, though the survival difference in G4 subgroup was not statistically significant. This might be attributed to the great discrepancy in the two comparing groups (166 cases of G4 glioma presented high expression of *ISG20*, while only 6 cases of G4 glioma expressed low level of ISG20). According to the fifth edition of the World Health Organization classification of tumors of the central nervous system (WHO CNS5), the primary genetic markers for gliomas are IDH mutation status, 1p19q codeletion, H3F3A alterations, ATRX gene mutations, MGMT promoter methylation status, loss of CDKN2A, and EGFR amplification, a combined gain of chromosome 7 and loss of chromosome 10, and TERT promoter pathogenic variants [29, 30]. The WHO CNS5 has substantially changing the classification of gliomas due to the increasing focus on molecular characteristics. Above results would inspire the further exploration of issues regarding the association of *ISG20* and other glioma molecular biomarkers.

To further clarify the functional role of ISG20 in gliomas, we performed an enrichment analysis based on DEGs between the high and low *ISG20* expression groups. We identified many terms associated with neuroplasticity, including synaptic signaling, neuron-to-neuron synapse, neurotransmitter receptor activity, and neuroactive ligand-receptor interaction. We found that the DEGs were enriched in inflammatory response, immune receptor activity, cytokine-cytokine receptor interaction, and leukocyte activation. Furthermore, enrichment analysis also indicated that *ISG20* was associated with ECM, ECM receptor interaction, and regulation of cell adhesion and activation. These results imply that ISG20 is associated with normal physiological processes in the CNS and pathophysiological processes in glioma, especially the immuno-inflammatory response and ECM function.

In recent years, our understanding of the epigenetic mechanisms involved in tumor pathology has improved greatly. DNA and histone modifications, such as methylation, demethylation, acetylation, and deacetylation, can lead to the up-regulation of oncogenic genes, as well as the suppression of tumor suppressor genes [31]. Cheng et al [32]. reported that hypermethylation of *ISG20* in kidney renal clear cell carcinoma and pancreatic adenocarcinoma tumor tissues is correlated with higher expression of *ISG20*, suggesting that methylation of *ISG20* may not underlie its overexpression. Gene expression can also be modified on a post-transcriptional level by microRNAs that contribute to carcinogenesis [33]. Alsheikh et al [18]. found that disruption of STAT5A and NMI signaling axis keeps a check on *ISG20* expression via miR-17–92 cluster, contributing to the ISG20-driven metastasis of mammary tumors. Protein post-translational modifications are enzymatic or nonenzymatic chemical reactions featuring the addition of chemical moieties, peptides or sugars to specific amino acid side chains, which makes a gene correspond to more than one protein and gives more complexity to the life process [34]. Protein phosphorylation is the most abundant and common protein post-translational in the human body, and is usually the first wave of protein modifications in response to intracellular and extracellular signaling [35]. Further analysis to unveil the mechanism underlying the abnormal expression of ISG20 and phosphorylation of the downstream immune proteins activated by ISG20 is of great interest in the future study.

Various immune cells, including T cells, B cells, NK cells, macrophages, and dendritic cells, mediate immunological response [36]. These immune components infiltrate the TME and either directly destroy tumor cells or facilitate their evasion of immunological surveillance [36]. Dysregulation of immune related genes and abnormal infiltration of immune cells in TME can serve as novel predicting biomarkers of human cancers. For example, CD276 and the gene signature composed of GATA3 and LGALS3 enable prognosis prediction of GBM [37]. Besides, correlation between lower balance of Th2 helper T-cells and expression of PD-L1/PD-1 axis genes enables prognostic prediction in patients with GBM [38]. Considering the correlation between high *ISG20* expression and poor prognosis, we hypothesized that ISG20 enhances tumor immune evasion. To determine the precise immune function of ISG20, we analyzed the correlation coefficient between *ISG20* and the 22 types of immune cells infiltrating the glioma TME. As anticipated, *ISG20* was positively correlated with inhibitory immune cells, such as M2 macrophages and Tregs. M2 macrophages are derived from myeloid cells and play a more important role in tumor support than pro-inflammatory M1 macrophages [39, 40]. We confirmed the above bioinformatics findings by visualizing the cellular co-localization of ISG20 and the M2 macrophage marker CD163 in glioma specimens using immunofluorescence analysis. Moreover, we investigated the correlation between *ISG20* and a series of immune checkpoints as well as the effectiveness of immune checkpoint blockage therapy. We showed that *ISG20* was positively correlated with inhibitory immune checkpoints and the treatment efficacy of PD1 blockage. Combination therapy is the mainstream treatment for gliomas in the future [41, 42]. Neurosurgery, radiotherapy, chemotherapy, targeted therapy, and immunotherapy will be integrated into comprehensive glioma treatment. These results demonstrate that ISG20 plays a pivotal role in establishing an immunosuppressive TME through M2 macrophages in glioma and might be a promising biomarker for the treatment efficacy of immunotherapy.

## Conclusions

ISG20 is expressed in M2 macrophages and can serve as a novel indicator for predicting malignant phenotypes and clinical prognosis in glioma patients. Our study provides insights into the cellular and molecular basis of the glioma immune microenvironment and identifies novel therapeutic targets for immunotherapy. Prospective clinical investigations and in vitro and in vivo studies of ISG20 function and relevant pathways are required to confirm and extend the findings presented here.

## Supplementary Information

**Additional file 1. **Table S1

**Additional file 2. **Table S2

**Additional file 3. **Table S3

**Additional file 4. **Table S4

**Additional file 5. **Table S5

**Additional file 6. **Table S6

**Additional file 7. **Table S7

**Additional file 8. **Table S8

**Additional file 9. **Supplementary fig 1

## Data Availability

The datasets generated or analyzed in this study are available in open access databases. In this study we used the following databases for analysis, data acquisition and visualization: HPA (http://www.proteinatlas.org), ESTIMATE (https://bioinformatics.mdanderson.org/estimate/), GENT2 (http://gent2.appex.kr/gent2/), UCSC Xena (http://xena.ucsc.edu/), Metascape (http://metascape.org), TCIA (https://tcia.at/patients), TIMER2.0 (http://timer.comp-genomics.org/). All data are available from the corresponding author upon reasonable request.

## Abbreviations

GENT2: Gene Expression database of Normal and Tumor tissues 2
TAMs: Tumor-associated macrophages
Tregs: Regulatory T cells
ECM: Extracellular matrix
IPS: Immunophenoscore
DEGs: Differentially expressed genes
CNS: Central nervous system
TME: Tumor microenvironment
ECM: Extracellular matrix
KEGG: Kyoto Encyclopedia of Genes and Genomes

## References

[1] Lapointe S, Perry A, Butowski NA (2018). Primary brain tumours in adults. Lancet.

[2] Ostrom QT, Gittleman H, Liao P, Vecchione-Koval T, Wolinsky Y, Kruchko C (2017). CBTRUS Statistical Report: Primary brain and other central nervous system tumors diagnosed in the United States in 2010–2014. Neuro Oncol.

[3] Stupp R, Mason WP, van den Bent MJ, Weller M, Fisher B, Taphoorn MJ (2005). Radiotherapy plus concomitant and adjuvant temozolomide for glioblastoma. N Engl J Med.

[4] Yabo YA, Niclou SP, Golebiewska A (2022). Cancer cell heterogeneity and plasticity: A paradigm shift in glioblastoma. Neuro Oncol.

[5] Chen Z, Hambardzumyan D (2018). Immune Microenvironment in Glioblastoma Subtypes. Front Immunol.

[6] Perus LJM, Walsh LA (2019). Microenvironmental Heterogeneity in Brain Malignancies. Front Immunol.

[7] Gongora C, David G, Pintard L, Tissot C, Hua TD, Dejean A (1997). Molecular cloning of a new interferon-induced PML nuclear body-associated protein. J Biol Chem.

[8] Gongora C, Degols G, Espert L, Hua TD, Mechti N (2000). A unique ISRE, in the TATA-less human Isg20 promoter, confers IRF-1-mediated responsiveness to both interferon type I and type II. Nucleic Acids Res.

[9] Weiss CM, Trobaugh DW, Sun C, Lucas TM, Diamond MS, Ryman KD (2018). The Interferon-Induced Exonuclease ISG20 Exerts Antiviral Activity through Upregulation of Type I Interferon Response Proteins. mSphere.

[10] Imaizumi T, Mechti N, Matsumiya T, Sakaki H, Kubota K, Yoshida H (2008). Expression of interferon-stimulated gene 20 in vascular endothelial cells. Microbiol Immunol.

[11] Pentecost BT (1998). Expression and estrogen regulation of the HEM45 MRNA in human tumor lines and in the rat uterus. J Steroid Biochem Mol Biol.

[12] Deymier S, Louvat C, Fiorini F, Cimarelli A (2022). ISG20: an enigmatic antiviral RNase targeting multiple viruses. FEBS Open Bio.

[13] Degols G, Eldin P, Mechti N (2007). ISG20, an actor of the innate immune response. Biochimie.

[14] Gao M, Lin Y, Liu X, Li Y, Zhang C, Wang Z (2019). ISG20 promotes local tumor immunity and contributes to poor survival in human glioma. Oncoimmunology.

[15] Miyashita H, Fukumoto M, Kuwahara Y, Takahashi T, Fukumoto M (2020). ISG20 is overexpressed in clinically relevant radioresistant oral cancer cells. Int J Clin Exp Pathol.

[16] Xu T, Ruan H, Gao S, Liu J, Liu Y, Song Z (2020). ISG20 serves as a potential biomarker and drives tumor progression in clear cell renal cell carcinoma. Aging (Albany NY).

[17] Lin SL, Wu SM, Chung IH, Lin YH, Chen CY, Chi HC (2018). Stimulation of Interferon-Stimulated Gene 20 by Thyroid Hormone Enhances Angiogenesis in Liver Cancer. Neoplasia.

[18] Alsheikh HAM, Metge BJ, Pruitt HC, Kammerud SC, Chen D, Wei S (2021). Disruption of STAT5A and NMI signaling axis leads to ISG20-driven metastatic mammary tumors. Oncogenesis.

[19] Xiong H, Zhang X, Chen X, Liu Y, Duan J, Huang C (2021). High expression of ISG20 predicts a poor prognosis in acute myeloid leukemia. Cancer Biomark.

[20] Park SJ, Yoon BH, Kim SK, Kim SY (2019). GENT2: an updated gene expression database for normal and tumor tissues. BMC Med Genomics.

[21] Li T, Fu J, Zeng Z, Cohen D, Li J, Chen Q (2020). TIMER2.0 for analysis of tumor-infiltrating immune cells. Nucleic Acids Res..

[22] Charoentong P, Finotello F, Angelova M, Mayer C, Efremova M, Rieder D (2017). Pan-cancer Immunogenomic Analyses Reveal Genotype-Immunophenotype Relationships and Predictors of Response to Checkpoint Blockade. Cell Rep.

[23] Ogata H, Goto S, Sato K, Fujibuchi W, Bono H, Kanehisa M (1999). KEGG: Kyoto Encyclopedia of Genes and Genomes. Nucleic Acids Res.

[24] Zhou Y, Zhou B, Pache L, Chang M, Khodabakhshi AH, Tanaseichuk O (2019). Metascape provides a biologist-oriented resource for the analysis of systems-level datasets. Nat Commun.

[25] Subramanian A, Tamayo P, Mootha VK, Mukherjee S, Ebert BL, Gillette MA (2005). Gene set enrichment analysis: a knowledge-based approach for interpreting genome-wide expression profiles. Proc Natl Acad Sci U S A.

[26] Khan F, Pang L, Dunterman M, Lesniak MS, Heimberger AB, Chen P (2023). Macrophages and microglia in glioblastoma: heterogeneity, plasticity, and therapy. J Clin Invest..

[27] Ostrom QT, Gittleman H, Stetson L, Virk SM, Barnholtz-Sloan JS (2015). Epidemiology of gliomas. Cancer Treat Res.

[28] Weller M, van den Bent M, Preusser M, Le Rhun E, Tonn JC, Minniti G (2021). EANO guidelines on the diagnosis and treatment of diffuse gliomas of adulthood. Nat Rev Clin Oncol.

[29] Louis DN, Perry A, Wesseling P, Brat DJ, Cree IA, Figarella-Branger D (2021). The 2021 WHO Classification of Tumors of the Central Nervous System: a summary. Neuro Oncol.

[30] Wen PY, Packer RJ (2021). The 2021 WHO Classification of Tumors of the Central Nervous System: clinical implications. Neuro Oncol.

[31] Mazor T, Pankov A, Song JS, Costello JF (2016). Intratumoral Heterogeneity of the Epigenome. Cancer Cell.

[32] Cheng J, Fu J, Tan Q, Liu Z, Guo K, Zhang L (2022). The regulation of ISG20 expression on SARS-CoV-2 infection in cancer patients and healthy individuals. Front Immunol.

[33] Pekarek L, Torres-Carranza D, Fraile-Martinez O, García-Montero C, Pekarek T, Saez MA (2023). An Overview of the Role of MicroRNAs on Carcinogenesis: A Focus on Cell Cycle, Angiogenesis and Metastasis. Int J Mol Sci..

[34] Wang H, Yang L, Liu M, Luo J (2023). Protein post-translational modifications in the regulation of cancer hallmarks. Cancer Gene Ther.

[35] Bilbrough T, Piemontese E, Seitz O (2022). Dissecting the role of protein phosphorylation: a chemical biology toolbox. Chem Soc Rev.

[36] Yu M, Chang Y, Zhai Y, Pang B, Wang P, Li G (2022). TREM2 is associated with tumor immunity and implies poor prognosis in glioma. Front Immunol.

[37] Takashima Y, Kawaguchi A, Hayano A, Yamanaka R (2019). CD276 and the gene signature composed of GATA3 and LGALS3 enable prognosis prediction of glioblastoma multiforme. PLoS ONE.

[38] Takashima Y, Kawaguchi A, Kanayama T, Hayano A, Yamanaka R (2018). Correlation between lower balance of Th2 helper T-cells and expression of PD-L1/PD-1 axis genes enables prognostic prediction in patients with glioblastoma. Oncotarget.

[39] Gabrusiewicz K, Rodriguez B, Wei J, Hashimoto Y, Healy LM, Maiti SN (2016). Glioblastoma-infiltrated innate immune cells resemble M0 macrophage phenotype. JCI Insight..

[40] Müller S, Kohanbash G, Liu SJ, Alvarado B, Carrera D, Bhaduri A (2017). Single-cell profiling of human gliomas reveals macrophage ontogeny as a basis for regional differences in macrophage activation in the tumor microenvironment. Genome Biol.

[41] McKinnon C, Nandhabalan M, Murray SA, Plaha P (2021). Glioblastoma: clinical presentation, diagnosis, and management. BMJ.

[42] Wen PY, Weller M, Lee EQ, Alexander BM, Barnholtz-Sloan JS, Barthel FP (2020). Glioblastoma in adults: a Society for Neuro-Oncology (SNO) and European Society of Neuro-Oncology (EANO) consensus review on current management and future directions. Neuro Oncol.

