# Supplementary material for: Integrated bioinformatics analysis and experimental validation reveal ISG20 as a novel prognostic indicator expressed on M2 macrophage in glioma
Source: BMC Cancer. 2023 Jun 28;23:596. doi: 10.1186/s12885-023-11057-0 (PMC10303331; doi:10.1186/s12885-023-11057-0)
Supplement: Supplementary file 1 — Additional file 1. Table S1 [file 12885_2023_11057_MOESM1_ESM.docx]

**Table S1.** Baseline characteristics of TCGA glioma patients included in this study.

| Characteristics | Number of cases (N = 697) |
| --- | --- |
| **Age, years** |  |
| < 55 | 472 |
| >= 55 | 223 |
| NA | 2 |
| **Gender** |  |
| Male | 398 |
| Female | 297 |
| NA | 2 |
| **IDH mutation** |  |
| Wild-type | 244 |
| Mutant | 441 |
| NA | 12 |
| **1p19q codeletion** |  |
| Codeleted | 172 |
| Non-codeleted | 510 |
| NA | 15 |
| **MGMT methylation** |  |
| Methylated | 492 |
| Unmethylated | 166 |
| NA | 39 |
| **WHO grade** |  |
| G2 | 258 |
| G3 | 271 |
| G4 | 166 |
| NA | 2 |
| **Histology** |  |
| O | 198 |
| AO | 134 |
| A | 197 |
| GBM | 167 |
| NA | 1 |
| **Primary therapy outcome** |  |
| CR | 135 |
| PR | 65 |
| PD | 116 |
| SD | 138 |
| NA | 243 |
| **Survival status** |  |
| Dead | 266 |
| Alive | 429 |
| NA | 2 |

O: oligodendroglioma, AO: anaplastic oligodendrocytoma, A: astrocytoma, GBM: glioblastoma, CR: complete response, PR: partial response, PD: progressive disease, SD: stable disease, NA: not available.
