# Supplementary material for: Integrated bioinformatics analysis and experimental validation reveal ISG20 as a novel prognostic indicator expressed on M2 macrophage in glioma
Source: BMC Cancer. 2023 Jun 28;23:596. doi: 10.1186/s12885-023-11057-0 (PMC10303331; doi:10.1186/s12885-023-11057-0)
Supplement: Supplementary file 3 — Additional file 3. Table S3 [file 12885_2023_11057_MOESM3_ESM.docx]

**Table S3.** Clinical information of 28 glioma samples

| No. | Age | Gender | WHO grade | Histological type |
| --- | --- | --- | --- | --- |
| 1 | 40 | Male | G2 | Diffuse astrocytoma |
| 2 | 22 | Female | G2 | Pleomorphic xanthoastrocytoma |
| 3 | 45 | Male | G2 | Diffuse astrocytoma |
| 4 | 52 | Male | G2 | Diffuse astrocytoma |
| 5 | 62 | Male | G2 | Oligodendrocytoma |
| 6 | 34 | Female | G2 | Oligodendrocytoma |
| 7 | 24 | Female | G2 | Diffuse astrocytoma |
| 8 | 33 | Male | G3 | Astrocytoma |
| 9 | 54 | Male | G3 | Anaplastic astrocytoma |
| 10 | 59 | Male | G3 | Anaplastic astrocytoma |
| 11 | 61 | Male | G4 | Glioblastoma |
| 12 | 50 | Female | G4 | Glioblastoma |
| 13 | 67 | Female | G4 | Glioblastoma |
| 14 | 59 | Male | G4 | Glioblastoma |
| 15 | 59 | Male | G4 | Glioblastoma |
| 16 | 67 | Male | G4 | Glioblastoma |
| 17 | 56 | Female | G4 | Glioblastoma |
| 18 | 42 | Female | G4 | Diffuse astrocytoma |
| 19 | 53 | Female | G4 | Glioblastoma |
| 20 | 58 | Female | G4 | Glioblastoma |
| 21 | 34 | Female | G4 | Diffuse astrocytoma |
| 22 | 51 | Female | G4 | Glioblastoma |
| 23 | 34 | Male | G4 | Glioblastoma |
| 24 | 46 | Male | G4 | Glioblastoma |
| 25 | 34 | Female | G4 | Glioblastoma |
| 26 | 47 | Female | G4 | Glioblastoma |
| 27 | 59 | Male | G4 | Glioblastoma |
| 28 | 62 | Male | G4 | Glioblastoma |
