# Supplementary material for: Integrated bioinformatics analysis and experimental validation reveal ISG20 as a novel prognostic indicator expressed on M2 macrophage in glioma
Source: BMC Cancer. 2023 Jun 28;23:596. doi: 10.1186/s12885-023-11057-0 (PMC10303331; doi:10.1186/s12885-023-11057-0)
Supplement: Supplementary file 9 — Additional file 9. Supplementary fig 1 [file 12885_2023_11057_MOESM9_ESM.docx]

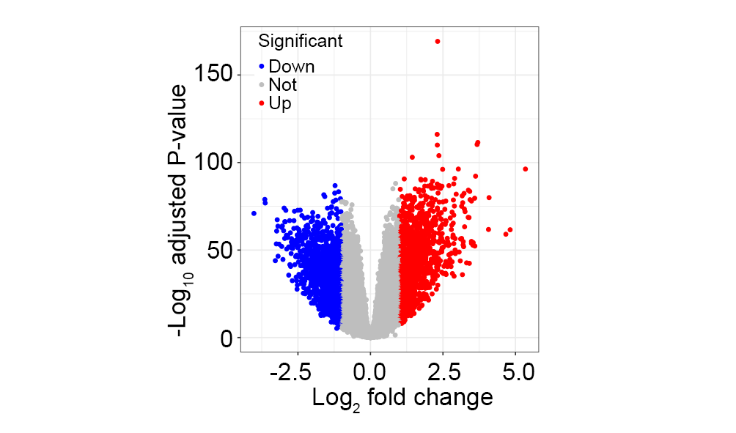


**Figure S1.** Differential expressed genes between the glioma patient subgroups classified by the median expression level of ISG20. The screening criteria were log_2_ |fold change| ≥ 1 and adjusted P-value < 0.05.
